# Sir Henry Burdett, K.C.B., K.C.V.O. 1847–1920

**Published:** 1920-05-08

**Authors:** 


### SIR HENRY BURDETT, K.C.B., K.C.V.O.

### 1847—1920.

***As was announced in the later edition of "The Hospital" on May 1, Sir Henry Burdett, after an illness of some months, passed away peacefully at his residence, 43 Porchester Terrace, on Thursday, April 29, in the seventy-fourth year of his age. The funeral service was held on May 3 at Holy Trinity, Paddington, and his last resting-place is at Worthing, where his mother was buried.***

HENRY CHARLES BURDETT, who was born on March 18, 1847, at Broughton, Northants, was the son of the Rev. Halford Robert Burdett, of Gilmorton, a village in Leicestershire, where in previous generations members of the family had often held the living. His mother, who before her marriage was a Miss Brailsford, came of a Lincolnshire family, and it was with her relatives at Barkwith and Toft Grange in that county that he used to spend his holidays. To the last he had happy recollections of these visits to what, from all accounts, seems to have been a typical country home of the period before motor-cars, when the "open-air life" meant outdoor existence in any weather.

His schoolmaster was Mr. George Ager, of Aylsham. Norfolk, and he was not only a master, but also an enduring friend. The pupil cultivated his friendship with Dr. Ager, for at least until his thirty-third year correspondence was maintained between the two. Though we shall be anticipating, it seems proper to insert here two extracts from Dr. Ager's side of the correspondence. The first extract is from a letter dated June 3, 1879: "And now, Burdett, for yourself from myself. We have only met three times lately. Yet, though I had only seen you once and for a very short time during that visit, that [visit] induced me to think that you will (*sic*) win as high or a higher place than any old Aylsham boy. I only noticed one thing that may prove a stumbling stone, and would not mention it, but that it *was mine*, and much I rue it. However, of this some other time." In the second letter, dated June 12, 1879, Dr. Ager wrote: "The stumbling-block, then, is . . . . a somewhat vehement, impetuous desire to put your foot *firmly* upon anything that gives offence, annoyance, or injury (in any way). I do not think that I can give any other explanation of the matter."

In 1863, on the death of his father, any previous plan, such as that the son should follow the father to Emmanuel College, Cambridge, was interrupted. As Mr. T. P. O'Connor's words on another page suggest, if "the country parsonage" produces sterling qualities, these usually reside less in money than in brains. He proceeded, therefore, to Birmingham, where he apprenticed himself to banking. In 1868 he was appointed superintendent and secretary at Queen's Hospital, Birmingham, and it was while he was there that the study of medicine attracted him. Only a few years ago, when by chance in the secretary's room in Queen's Hospital, Birmingham, the present writer saw a likeness of Sir Henry on the wall. To an interested observer it was unexpected, and excited many recollections, and reflection became busy with the past, for the years 1868-74, when Sir Henry was in Birmingham, have become of interest to many people not connected in any way with hospital life. The star of Mr. Joseph Chamberlain was already rising above the horizon of the Midland city, which was also, like the sixties generally, full of an eager municipal life. Locally, under Mr. Chamberlain's influence, municipal politics were engaging energy and attention. Two such personalities as those of Joseph Chamberlain and Henry Burdett, living in the same provincial city, equally poised in energy and ambition, could not but enter one another's sphere. The fact of their acquaintance can be but noted here, though a long chapter might be made of the incidents connected with it.

In regard to his work for Queen's Hospital during his superintendentship, there is room only for two facts—first, on his appointment to the post of superintendent at Queen's Hospital in 1868 the annual income of Queen's Hospital was £7,000. When he resigned this post in 1874 in order to become house governor of the Dreadnought Seamen's Hospital, Greenwich, his efforts had raised the income of Queen's Hospital to £13,000 a year. Secondly, in the same period and by the same controlling hand the medical school of Queen's College and the Queen's Hospital, hitherto independent, if associated, organisations, had merged into one body under one authority and with one inclusive title. On the occasion of his departure from Birmingham in January 1874 he was presented with a clock by the past and present resident officers of the Queen's Hospital, Birmingham. In May he received a gold watch and chain, the former from the town of Birmingham and the latter from the working men engaged in rebuilding the Queen's Hospital, the latter of whom were the founders of the Hospital Saturday Fund there, which was one of the first and most successful of all similar funds which Sir Henry co-operated with Mr. Sampson Gamgee, F.R.C.S.Edin., in organising and making successful originally. In 1875, he married Helen, daughter of Gay Shute, F.R.C.S., a surgeon in practice at Greenwich.

In 1881 Sir Henry Burdett was appointed to the office of Secretary to the Share and Loan Department of the Stock Exchange, and the ensuing years were marked by many of his most remarkable achievements. Notwithstanding the engrossing labours which fell upon him in the entire reorganisation of this department, he was able to undertake a dozen schemes of importance and to inaugurate a dozen enterprises, the conception and execution of any one of which would have brought enough work and more to a man of ordinary energy. It would be possible to write several long accounts

**Biography.—(*continued*).**

of Sir Henry's separate activities during this period, none of which now contain material used in any of the others. It marked a prodigious output which would be difficult to parallel among his contemporaries. Now and almost to the end of his life his habit was to take only four or five hours of sleep and devote the rest of the night to work.

To give a list of the books written and published during these years is to convey the impression that he spent his whole time in authorship. The monumental "Hospitals of the World," with portfolio of plans, the first volume of which appeared in 1891 and the fourth in 1893, occupied some years and proved a harder task than he had reckoned for. It was soon recognised as the leading authority on the subject, and is a mine of information of a kind which was previously inaccessible in any collected form. Numerous hand-books on subjects relating to financial matters, to hospitals and nurses, preceded and followed this important work, each inspired by the desire to form a reliable guide to a previously unexplored subject.

Sir Henry Burdett, jointly with Professor de Chaumont, was Editor of the first three volumes of the Proceedings of the Sanitary Institute of Great Britain, and served as a member of the Council from 1879 to 1883.

His contributions to the proceedings were numerous and varied. The following are the principal heads under which his papers may be grouped:—(*a*) The Administration and Hygiene of British Hospitals; (*b*) The Dwellings of the Middle Classes; (*c*) The Examination of Sanitary Officers; (*d*) The Necessity and Importance of Mortuaries; (*e*) The Unhealthiness of Public Institutions; (*f*) The Dwellings of the Poor in Large Towns; (*g*) A Report upon the Polluted Condition of the Thames; and (*h*) Miscellaneous Papers.

It was in accord with the vogue of the monthlies serial to provide channels into which week by week and year by year the streams of reliable information and sound criticism which he spent so much of his life in accumulating could be turned. He loved the periodical form, for he lived half his time in the future, and a form of expresion which lent itself to continual revision, a continual aggregation of freshly observed facts, a rapid adjustment to new ideas, was fitted to show his powers to the greatest advantage. It was in the year 1886 that he started The Hospital, a paper which he regarded from its inception till his last hour with peculiar affection, and into which he threw his most cherished convictions, his ripest wisdom, week by week, till the end. Within the pages of The Hospital lies hidden his true autobiography. By 1907 the nursing supplement of The Hospital, the *Nursing Mirror*, had gradually grown so popular as to demand a separate existence. As the oldest nurses' paper it has held its place ever since, and its influence has been made manifest in an emphatic manner on several occasions. "Burdett's Hospitals and Charities—the Yearbook of Philanthropy" started on its course in 1889, and gradually became a most valuable ally of all progressive hospital managers. It was consulted habitually by King Edward, and an asterisk affixed in its pages against an organisation which had refused information led to an enquiry which ended with the withdrawal of the King's name as patron. Closely linked with this work is the "Uniform System of Accounts," originated by Sir Henry Burdett in 1869, and now almost universally adopted by hospitals in this country and the Colonies. The system forms the basis of the scheme to which hospitals participating in the benefits of the King's Fund are compelled to conform, and has had a salutary effect in promoting economy wherever it is introduced.

It was peculiarly in the genius of Sir Henry Burdett to persuade and even, as it were, to compel men to come together and make association to carry out a common purpose. When he saw things which needed doing in the interests of the public he was able with a sure instinct to bring together the right people and get them harnessed, willing or unwilling, to the good work.

In this way he was instrumental in founding the Home Hospitals' Association, the outcome of which was Fitzroy House, the first pay hospital in London and the precursor of pay-wards in many directions. It was he who founded the Association of Hospital Managers and Officials, and, as mentioned above, he was one of the original members of the Council of the Sanitary Institute of Great Britain. He was able to induce even nurses, at that time unorganised and unbusinesslike, to combine for their own interests, and by the formation of the Nurses' Co-operation he opened up a new realm of lucrative employment to the modern generation of private nurses.

He had much to do with the formation of the Metropolitan Ambulance Association, and aided forcefully to wipe out the reproach resting on London municipalities of having provided no adequate assistance for victims of accident or sudden illness. Largely owing to his unremitting efforts easily accessible ambulances were at length provided in every district. There were four great Funds with which his name will always be associated, and his influence with the City had much to do with the success of all four.

When the Hospital Sunday Fund was started in 1873, on the model of the Fund started in Birmingham by Canon Miller, Sir Henry was invited to sit on the Council, and he speedily justified the honour which had been done him. From the first he was one of the most zealous organisers enlisted in its service, and he had the gratification, in 1896, of raising, through the special Hospital Sunday Supplement of The Hospital, no less than £20,000 for its coffers.

The Royal National Pension Fund for Nurses owes its origin to Sir Henry's passionate determination that nurses should eat of the fruit of their labours and not find themselves cast aside without protection when working days were over. The story of how the Fund was founded is too long to

**Biography**—(*continued*).

tell here. It must suffice to say that the sum of £20,000 was raised, which formed the nucleus of a Donation Bonus Fund. Within ten years 5,000 nurses had joined the Fund, to-day there are 2,550 receiving pensions of £67,000 a year, while the total investments amount to over £2,000,000. There has been no greater factor in assuring the prosperity of the nursing profession.

The Prince of Wales's Hospital Fund, later known as King Edward VII. Hospital Fund for London, had no more devoted champion than Sir Henry, who had a life-long faith in the generosity of the public towards the hospitals. He saw that while various hospitals had been able to enlist the support of distinct groups of influential people, there was no central hospital fund with prestige sufficient to form a guarantee to donors that their gifts and legacies would be wisely bestowed. Sir Henry's forecast of an income of £100,000 a year was considered fantastic when the Fund was founded. He lived to see the sum of £230,000 distributed among London Hospitals in one year. Always alive to the value of the small giver, Sir Henry assisted to start the League of Mercy, which has already interested many thousands in hospital work who had scarcely heard of it before and has brought in to date upwards of £293,000 for the benefit of the sick poor.

All this time Sir Henry was ceaselessly bombarded with requests for information, advice, and assistance. He was called on to adjudicate in many a dispute and deadlock, especially on matters relating to hospitals and nursing administration. Whenever anything went hopelessly wrong in an institution he was appealed to, and apart from visits of inspection arising out of such difficulties, he found time to make exhaustive visits of investigation into almost every hospital in the country. Hundreds of philanthropists were eager to pick his brains. He was for ever being called on to furnish plans for expansion, for retrenchment, for new projects, and he lent an attentive ear to every and all. He drew up countless reports and recommendations; he infused a new live spirit into whatever he touched, and was a frequent source of inspiration to harassed managers and administrators.

He had an extraordinarily clear vision, physically and mentally. He was an adept at laying foundations. All his chief enterprises are going-concerns, abreast of the times, capable of adaptation to changing conditions. He welcomed ideas wherever he found them, but suffered fools less gladly, perhaps, than was prudent. Intensely individual, intensely pugnacious where principles were concerned, intensely loyal, he fought his way through these years of stress, and when the battle-field is surveyed the solid masonry of the barriers he raised for his fellows against suffering and despair stand out clear and strong against the sky. They were built to endure.

After retiring from the Share and Loan Department of the Stock Exchange (1898) he became associated with important business undertakings. This did not in the least imply any slackening of his devotion to institutional affairs, or his interest in the financial problems entailed by the voluntary hospital. It was in the early years of the new century that he became greatly engrossed in arousing public interest in the League of Mercy, of which he was Honorary Treasurer from its foundation until 1915. In the winter of 1902-03 he addressed a large number of meetings in support of the infant League, and succeeded in enlisting widespread support for it; all this in addition to his other work. About this time also (1903) he threw himself into the proposals in connection with the rebuilding of St. Bartholomew's Hospital; the raising of £10,000 for the reconstruction of Northampton General Infirmary (now Hospital), by the Governors of which he was called in in 1901 to advise how best to bring the institution up to date; the rebuilding of the Glasgow Royal Infirmary, the plans for which were begun in 1908; and the opening of the New Pension Fund Building of the Royal National Pension Fund in Buckingham Street in the same year, when Sir Henry received the K.C.V.O.

For a long time it had been found necessary to print a separate supplement to The Hospital devoted exclusively to nursing matters, and in 1907 the decision was taken to issue this as a separate weekly newspaper—*The Nursing Mirror*. Of this, as of the parent newspaper, Sir Henry was editor-in-chief. The new venture was immediately successful, partly owing to the loyalty with which the Editor was served by his subordinates, partly owing to the wise policy of inviting contributions from as wide a circle of readers as cared to respond, and partly owing to the constant care with which it was supervised. Of *The Nursing Mirror*, as of The Hospital, he remained Editor from the first number down to the day of his death.

In the years preceding the war the most notable of his activities, perhaps, was the series of visits to and reports upon hospitals of the United Kingdom. These reports, like those of a preceding series twenty years before, were published in this Journal and excited widespread interest. In the midst of all these manifold occupations Sir Henry found time for recreation. In earlier days lawn tennis, then boating, and, later still, billiards and croquet attracted him. Bird life was also one of his hobbies, and he was on the friendliest terms with the caged birds which he habitually kept in his workroom.

The war itself engrossed him very fully, and his ardent sympathies were instantly enlisted on behalf of the sufferers which it produced. When he was offered the opportunity of seeing for himself the work of the R.A.M.C. in France he accepted eagerly, feeling that at last he could be of direct service to the wounded by giving of his experience on their behalf. To his keen appreciation of after-war problems in the hospital world the pages of this Journal amply testify. To this work he devoted himself continuously up to the onset of his last illness.

